# Repeated caffeine intake suppresses cerebral grey matter responses to chronic sleep restriction in an A_1_ adenosine receptor-dependent manner: a double-blind randomized controlled study with PET-MRI

**DOI:** 10.1038/s41598-024-61421-8

**Published:** 2024-06-03

**Authors:** Yu-Shiuan Lin, Denise Lange, Diego Manuel Baur, Anna Foerges, Congying Chu, Changhong Li, Eva-Maria Elmenhorst, Bernd Neumaier, Andreas Bauer, Daniel Aeschbach, Hans-Peter Landolt, David Elmenhorst

**Affiliations:** 1 Centre for Chronobiology, University Psychiatric Clinics Basel, Wilhelm Kleinstr. 27, 4002 Basel, Switzerland; 2 Research Cluster Molecular and Cognitive Neurosciences, University of Basel, Basel, Switzerland; 3 Athinoula. A. Martinos Center for Biomedical Imaging, Department of Radiology, Massachussetts General Hospital, Harvard Medical School, Boston, USA; 4 Department of Sleep and Human Factors, Institute of Aerospace Medicine, German Aerospace Center, Cologne, Germany; 5 Institute of Pharmacology and Toxicology, University of Zurich, Zurich, Switzerland; 6 Sleep & Health Zurich, University Center of Competence, University of Zurich, Zurich, Switzerland; 7 Institute of Neuroscience and Medicine, INM-2, Forschungszentrum Jülich, Wilhelm-Johnen-Strasse, 52428 Jülich, North Rhine-Westphalia Germany; 8 Department of Neurophysiology, Institute of Zoology (Bio-II), RWTH Aachen University, Aachen, Germany; 9 Institute for Occupational, Social, and Environmental Medicine, RWTH Aachen University, Aachen, Germany; 10 Institute of Neuroscience and Medicine, INM-5, Forschungszentrum Jülich, Jülich, Germany; 11 Institute of Experimental Epileptology and Cognition Research, University of Bonn Medical Center, Bonn, Germany; 12 Multimodal Neuroimaging Group, Department of Nuclear Medicine, University Hospital Cologne, Cologne, Germany

**Keywords:** Sleep deprivation, Neurochemistry, Receptor pharmacology

## Abstract

Evidence has shown that both sleep loss and daily caffeine intake can induce changes in grey matter (GM). Caffeine is frequently used to combat sleepiness and impaired performance caused by insufficient sleep. It is unclear (1) whether *daily* use of caffeine could prevent or exacerbate the GM alterations induced by 5-day sleep restriction (i.e. *chronic* sleep restriction, CSR), and (2) whether the potential impact on GM plasticity depends on individual differences in the availability of adenosine receptors, which are involved in mediating effects of caffeine on sleep and waking function. Thirty-six healthy adults participated in this double-blind, randomized, controlled study (age = 28.9 ± 5.2 y/; F:M = 15:21; habitual level of caffeine intake < 450 mg; 29 homozygous C/C allele carriers of rs5751876 of *ADORA2A,* an A_2A_ adenosine receptor gene variant). Each participant underwent a 9-day laboratory visit consisting of one adaptation day, 2 baseline days (BL), 5-day sleep restriction (5 h time-in-bed), and a recovery day (REC) after an 8-h sleep opportunity. Nineteen participants received 300 mg caffeine in coffee through the 5 days of CSR (CAFF group), while 17 matched participants received decaffeinated coffee (DECAF group). We examined GM changes on the 2nd BL Day, 5th CSR Day, and REC Day using magnetic resonance imaging and voxel-based morphometry. Moreover, we used positron emission tomography with [^18^F]-CPFPX to quantify the baseline availability of A_1_ adenosine receptors (A_1_R) and its relation to the GM plasticity. The results from the voxel-wise multimodal whole-brain analysis on the Jacobian-modulated T1-weighted images controlled for variances of cerebral blood flow indicated a significant interaction effect between caffeine and CSR in four brain regions: (a) right temporal-occipital region, (b) right dorsomedial prefrontal cortex (DmPFC), (c) left dorsolateral prefrontal cortex (DLPFC), and (d) right thalamus. The post-hoc analyses on the signal intensity of these GM clusters indicated that, compared to BL, GM on the CSR day was increased in the DECAF group in all clusters  but decreased in the thalamus, DmPFC, and DLPFC in the CAFF group. Furthermore, lower baseline subcortical A_1_R availability predicted a larger GM reduction in the CAFF group after CSR of all brain regions except for the thalamus. In conclusion, our data suggest an adaptive GM upregulation after 5-day CSR, while concomitant use of caffeine instead leads to a GM reduction. The lack of consistent association with individual A_1_R availability may suggest that CSR and caffeine affect thalamic GM plasticity predominantly by a different mechanism. Future studies on the role of adenosine A_2A_ receptors in CSR-induced GM plasticity are warranted.

## Introduction

Caffeine is the most widely used psychoactive substance^1^. Given its efficacy in improving alertness^2^ and alleviating cognitive impairments caused by sleep deprivation^3^ or sleep restriction^4^, it is often consumed to combat drowsiness^5^. On the cerebral level, both acute sleep loss and daily caffeine intake can lead to a decrease in human grey matter (GM) volumes^6–10^ as measured by magnetic resonance imaging (MRI). It is unclear whether caffeine alleviates or exacerbates the GM changes induced by insufficient sleep.

Sleep deprivation^11^ and sleep restrictions^12–14^ impair brain structures and functionality in rodents. In humans, while the effects of *chronic* sleep restriction (CSR) on GM are unclear, inconsistent changes in brain structures have been observed following different sleep disruptions. One-night sleep restriction (i.e. measuring after a night of 3-h time in bed) led to cortical changes specifically in young age groups, including decreased GM in the thalamus, precuneus, and postcentral gyrus and increased GM in the insula^6^. However, multiple cortical and subcortical GM regions could also change in a non-linear fashion in a time course of 24- to 72 h during a total sleep deprivation^7,8^. Although it has been shown that one-month sleep restriction could lead to reduced white matter diffusivity^15^, GM plasticity after a longer sleep restriction remained to be investigated. On the other hand, chronic caffeine administration has been found to inhibit hippocampal neurogenesis and cell proliferation in adult rodents^16–18^, and *daily* caffeine intake was found to be associated with reduced cortical and hippocampal GM volume in humans^9,10^. We hypothesized that GM would be reduced after CSR, while a concomitant caffeine intake could exacerbate the CSR-induced GM reduction.

Effects of both caffeine and sleep loss are mediated in part by the adenosine system^19,20^. Partly serving as a byproduct of neuronal activity, the release of extracellular endogenous adenosine is increased throughout wakefulness^21^, and the binding of adenosine to the A_1_ adenosine receptor (A_1_R) leads to neural inhibition. Extended wakefulness, including partial or complete sleep deprivation can lead to increased levels of extracellular adenosine^22^ and upregulated A_1_R binding^23^. Caffeine, a nonselective adenosine receptor antagonist, enhances synaptic excitation^24^, thereby improving alertness^19^, enhancing vigilance^2^, and counteracting sleep the distributions of the genotype, together with age loss-induced cognitive decline^3,4^. The magnitude of cerebral and behavioral responses to caffeine and sleep loss can vary in association with the A_1_R availability^25–27^. Hence, we expect that one's baseline A_1_R binding potentials may also predict the magnitudes of GM plasticity induced by CSR and caffeine. In addition, both the sensitivity to caffeine effects on sleep^28^ as well as the distribution of A_1_R^29^ are known to be associated with the variant rs5751876 of the A_2A_ adenosine receptor (A_2A_R) encoding gene, *ADORA2A*. Hence, we matched the participant  groups by the distributions of the genotype, together with age, gender, body mass index, chrono type, and level of habitual caffeine intake^4^.

Since the vasoconstrictive effect of caffeine can render bias in morphology structure through altering perfusion^30^, we also quantified cerebral blood flow (CBF) as a covariate in the morphometry analysis. As an exploratory step, we also examined whether CBF was altered after chronic sleep restriction. Earlier studies reported inconsistent changes after different durations of sleep loss. One study reported a reduced absolute CBF (absCBF) in the attention network after *acute* sleep restriction (4 h in bed) in subjects with higher drowsiness but elevated absCBF in basal forebrain in those who remained alert^31^. Others studies reported reduced relative CBF (rCBF) in parahippocampus, fusiform, and prefrontal cortices^32^, while rCBF was increased in occipital cortices and insula^33^ after one-night sleep deprivation. A diffusion MRI study revealed that one month of sleep restriction (5.5 h in bed) could lead to a reduced diffusivity in the orbitofrontal gyri, superior occipital gyri, insula, and fusiform but increased diffusivity in supplementary motor area and cingulate gyrus^15^. How *chronic* sleep restriction affects CBF remains to be explored.

## Methods

The study took place at the :envihab research facility of the German Aerospace Center (Cologne, Germany). The study protocol was approved by the Ethics Committee of the regional Medical Board (Ärztekammer Nordrhein) and the German Federal Office for Radiation Protection. The study execution followed the declaration of Helsinki, and all participants have provided their informed consent voluntarily in a written form. We registered the study on German Clinical Trial Registry DRKS (04.04.2018; detailed protocol see https://drks.de/search/en/trial/DRKS00014379). All the participants have given informed consent in written form. The recruitment and data collection took place from year 2018 to 2020.

### Study procedure

**Figure 1:**
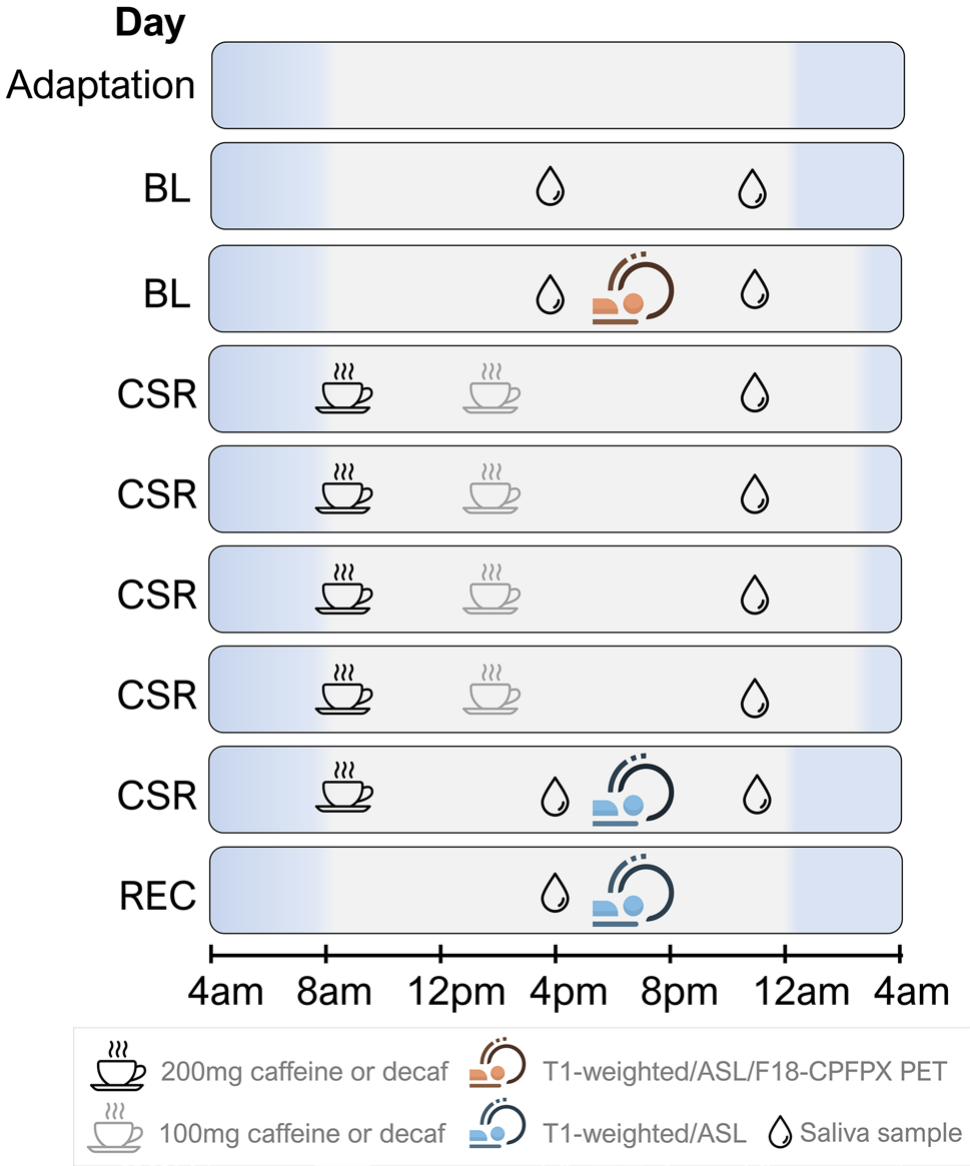
An overview of study protocol. The sleep restriction protocol consists of an adaptation day, two baseline days (BL), five sleep restriction days (CSR), and a recovery day (REC). The clock times of this protocol could be shifted according to the participant’s habitual bedtime, while the presented schedule is based on an 8 am–12 am wake-sleep schedule. On the nights of adaptation and the first BL days, participants had a full 8 h sleep (in blue shade). Starting from the second BL day throughout the first four CSR days, the bedtime was delayed by 3 h (i.e. 5-h time in bed). During the waking time (in while shade), participants were in constant luminance (~ 100 lx) during wakefulness. The coffee mug icons indicated the time when 19 participants received caffeine-containing coffee (CAFF group) and 17 received decaffeinated coffee (DECAF group). To monitor the caffeine levels, saliva samples were collected throughout the BL, CSR, and REC days (indicated by water drop icons). Three MRI scans took place in the afternoon of the second BL Day, the 5th CSR Day (average 7.0 ± 0.8 h after the last caffeine intake), and the REC Day (average 29.8 ± 4.3 h after the last caffeine intake), while for [^18^F]-CPFPX PET, we focused only on the baseline level measured on the second BL day.

In a double-blind randomized study, 36 healthy adults (aged 28.9 ± 5.2 y/o; females: males = 15:21) underwent 9 consecutive laboratory days in the following order: 1-day adaptation (8-h time in bed), 2-day baseline (BL, 8-h time in bed/day), 5-day CSR days (5-h time in bed/day), 1-day recovery with 8 h time in bed (REC; Fig. 1). All participants are non-smokers with a habitual intake level of caffeine < 450 mg. Twenty-nine participants were homozygous C/C allele carriers of ADORA2A rs5751876, an A_2A_R gene variant, while 6 were heterozygous C/T allele carriers and 1 was homozygous T/T allele carriers. Inclusion/exclusion criteria and demographic data by groups are reported in Supplement (under Supplementary methods and Table S2) and Ref.^27^).

All participants followed a one-week sleep satiation protocol (9 h in bed between 22:00 and 07:00 or between 23:00 and 08:00) prior to the start of the study. The compliance was monitored by daily self-reports and actimetry. Starting from the adaptation day, participants were in constant luminance (~ 100 lx) during wakefulness. Across the 5-day CSR, 19 of the 36 participants were assigned to the CAFF group and received daily coffee containing 200 mg (at 07:30 or 08:30) and 100 mg (at 14:00 or 15:00) of caffeine in the morning and at noon, respectively, while the other 17 participants assigned to the DECAF group received decaffeinated coffee^4^. [^18^F]-CPFPX PET-MRI acquisitions (Biograph mMR, Siemens) took place in the afternoon of the second BL Day, the 5th CSR Day (average 7.0 ± 0.8 h after the last caffeine intake), and the REC Day (average 29.8 ± 4.3 h after the last caffeine intake). To measure caffeine levels, salivary samples were collected repeatedly every evening (except for the adaptation day), as well as in the morning of the baseline days and before the PET scans (i.e. 1st and 2nd BL Day, 5th day of CSR and REC Day).

### Randomization and intervention

We used the Visual Basic for Applications program of the Institute for medical biostatistics, informatics and epidemiology (IMBIE), University Bonn, for a stratified block randomization with the factors age, sex and BMI. For the caffeine administration for the CAFF group, we gave 200 mg caffeine in the morning and 100 mg caffeine in the afternoon in the form of coffee beverage. Meanwhile, DECAF group received identical volumes of decaf coffee on the same schedule. The procedure and device for the preparation of the caffeine-containing coffee and decaf coffee were standardized in accordance with a detailed instruction of the manufacturer, Tchibo GmbH, Coffee Technology, Hamburg, Germany. Using high-quality, electric drip filter coffee machines (Tchibo type 5794 and 2855) and a standardized brewing protocol, a consistent amount of caffeine was yielded: per 200 g regular coffee contains 101 ± 0.6 mg caffeine, while per 200 g decaf coffee contains 2.4 ± 0.05 mg caffeine (data available in Ref.^4^). Accordingly, both CAFF and DECAF group received 400 g coffee beverage in the morning and 200 g in the afternoon. All research team members involved in data collection and statistical analyses stayed blind to the randomization until the end of the study.

To confirm a successful caffeine administration, we monitored the salivary caffeine concentration daily throughout the laboratory protocol (Fig. 1). Saliva samples were taken in the mornings on the two BL Days, the 5th CSR Day, and REC Day as well as the evenings of all BL and CSR Days using Salivette^®^ tubes (Sarstedt, Germany). Saliva samples were analyzed using an ultra-high-performance liquid chromatography system (Thermo Fisher, San Jose, CA), coupled with a linear ion trap quadrupole mass spectrometer 5500 (Sciex, Darmstadt, Germany; UHPLC-MS). For detailed protocol of sample preparation and UHPLC-MS analysis, please find the Method section of Ref.^4^.

### Power calculations

Statistical power was calculated based on the primary endpoint of the parent project of this study. There, we hypothesized that chronic partial sleep deprivation would lead to lasting changes in neuroreceptors in the striatum of the brain. This increase in adenosine receptor availability, as compared the level after the 5-day chronic sleep deprivation to the baseline, was expected to be reduced by the regular consumption of caffeine compared to placebo. The adenosine receptor availability would be quantified by distribution volume (VT).

Accordingly, the number of subjects was calculated based on this hypothesis using an unpaired T-test. We based the expected effect (d = 0.91) on the results of our earlier study^23^ for the mean VT in the striatum (mean = 1.0 ml/ml). A statistical power of 0.8 was aimed for in order to find a significant mean difference of 0.1 ml/ml, with an assumed standard deviation of 0.11 ml/ml. Statistical significance level was set at p = 0.05. As a result, a sample size of 20 people per group was targeted.

### Data acquisition and processing

After 3 dropouts in the DECAF group (85% completed) and 1 in the CAFF group (95% completed), we concluded the study with thirty-six subjects. Among the 36 subjects, one T1-weighted and Arterial Spin Labeling (ASL) images from the BL scan of a CAFF group subject were lost due to a technical reason on the scan day. In addition, during a CSR scan of another CAFF group subject as well as the REC scan of a DECAF group subject, the ASL images failed to be acquired.

In a series of stepwise analyses, we first identified the cortical changes in response to the main effect of CSR and the interaction between Caffeine and CSR. We conducted whole-brain multimodal voxel-wise morphometry (VBM) with linear mixed models, which allowed regressing out the variance of CBF voxel-to-voxel with ASL images. Next, we conducted a whole-brain exploratory analysis on ASL images to determine the main effect of CSR and the interaction effect between Caffeine and CSR on CBF. Lastly, we extracted the mean intensity of GM and mean CBF of the identified regions, as well as the binding potential (BP_ND_) of A_1_R in cortical and subcortical regions from all time points (i.e. baseline, CSR Day, and REC Day), respectively. Using the extracted values, we then conducted post-hoc analyses as well as examined the association between the GM and/or CBF responses and the A_1_R BP_ND_ with linear regressions.

#### T1-weighted images and GM intensity

Grey matter (GM) morphology was assessed by T1-weighted images using 3-dimensional magnetization-prepared rapid acquisition with gradient echo (3D MPRAGE; isotropic voxel 1 mm, TR = 2250 ms, TE = 3.03 ms). For the preprocessing of T1-weighted images, we used the “Segment Longitudinal Data” pipeline provided by CAT12 toolbox on SPM12 (University College London, London, UK), which enabled a co-registration with the mean of the three volumes collected from the three timepoints (i.e. BL, CSR, and REC) of each participant and was suitable for repeated measurements. Affine registration was conducted using the tissue probability map in SPM12, followed by the segmentation of brain tissues into grey matter, white matter, cerebrospinal fluid, as well as total intracranial volume. The procedure continues with the Jacobian-modulated normalization using an MNI (Montreal Neurological Institute, McGill University)-defined standard brain. Lastly, we smoothed the preprocessed data with Gaussian kernel of FWHM = 8 × 8 × 8 mm^3^.

Next, we identified the GM responses to CSR and the interaction effect between caffeine and CSR using a voxel-wise multimodal analysis in order to statistically control for the potential bias caused by caffeine-induced changes in brain perfusion^30,34,35^. We used VoxelStats toolbox^36^, with which we conducted linear mixed models on two types of co-registered 3D volumes, i.e. T1-weighted and ASL, through each corresponding voxel. In addition to CBF, we used age, sex, and total intracranial volume as regressors of no interests in the model. The statistical significance of the coefficients was acquired with permutation tests (5000 times) and defined by a threshold at cluster-level p_FDR_ < 0.001). To validate the novel toolbox VoxelStats and its voxel-to-voxel approach in tackling the bias of perfusion on morphometry, we compared the outcomes between controlling for voxel-wise CBF variances with VoxelStats and for global CBF variances with FMRIB Software Library (FSL 5.0; Oxford Center for Functional MRI of the Brain, United Kingdom) in the VBM analyses on T1-weighted images. Corresponding to the setup in Voxelstats, we used the “*randomise*” function (number of permutations = 5000) in FSL, and we used age, sex, and total intracranial volume as regressors as covariates. We present the statistical maps from VoxelStats and FSL in Supplementary method, Fig S1. In summary, the influence of CBF variances on the voxel and global level of the T1-weighted VBM analysis were strongly comparable. We believed that either (1) there was no significant contribution of CBF variances to the condition-driven GM changes (i.e. under caffeine or CSR) in general, or that (2) the within-subject comparison provided a good sensitivity to the condition-driven CBF variances and therefore well preserved the global variance to be reliably characterized on the voxel level. In any case, based on this precondition, we eventually decided to go for a voxel-wise approach as it could further take potential contributions of the regional CBF variances to the GM measurement into account, if any.

For the regions identified to be responsive to the interaction between caffeine and CSR, we further conducted a post-hoc analysis to determine the exact GM changes in each group in response to CSR using region-of-interest-based approach on R (R core team, Vienna, Austria). We first created masks of the responsive regions identified in the whole-brain analyses. Accordingly, we extracted mean signal intensity of these regions from all time points (BL, CSR, and REC) using FSL *fslmeants*, followed by a post-hoc analysis to determine the directions of GM responses in each group.

#### Cerebral blood flow

Cerebral blood flow (CBF) was measured by the sequence ASL (5 × 5 × 5 mm^3^, TR = 3500 ms, TE = 11 ms). For the preprocessing of ASL images, we used the standard pipeline on FSL. We used the first volume for the reference of motion correction as well as for the M0 calibration. We calculated the relative CBF maps with the acquired 40 tag-control pairs, followed by the quantification of absolute CBF using white matter as the reference tissue. Lastly, the absolute CBF maps were co-registered onto the T1-weighted images and MNI space.

Next, we conducted the whole-brain analysis on the absolute CBF maps with a GM mask using 2-way mixed effect ANOVA. Similar to the analysis on T1-weighted images, we also used nonparametric threshold-free cluster enhancement (number of permutations = 5000, cluster-level threshold p_FDR_ < 0.01) with the “*randomise*” function. Same as the GM analysis, a post-hoc analysis was carried out on the significant changes in response to Caffeination x CSR using an identical procedure in extracting the regional mean response and post-hoc analyses.

#### Cerebral A_1_R availability

We acquired the [^18^F]-CPFPX PET data (framing scheme: 4 × 60 s, 3 × 120 s, 18 × 300 s, 2.09 × 2.09 × 2.03 mm^3^) simultaneously with the magnetic resonance imaging (MRI) scan. The scan in total lasted 100 min. We adopted an intravenous bolus injection simultaneously with the start of the scan, followed by a constant infusion (15.9 ml/ 34.1 ml, Kbol = 55 min.) The eventual dose injected was 175.9 ± 21.8 MBq and the molar activity was 102.25 ± 72.08 GBq/µmol at the time of injection.

For the preprocessing of PET, we used the standard pipeline of PMOD Neuro Tool (v4.006; PMOD Technologies). We first conducted motion correction with the average of the PET data collected in the first 9 min. The matching PET image was co-registered with the corresponding preprocessed T1-weighted image and segmented into grey matter, white matter, and cerebrospinal fluid, followed by the spatial normalization based on the MNI space. Seventy volumes of interests (VOIs) were defined by the automated anatomical labelling template of MNI space. For the kinetic modeling we used PMOD Kinetics Tool (version 4.006; PMOD Technologies). We examined the time-activity curves (TACs) and used cerebellum as a reference region to calculate the BP_ND_ of each volume of interest. The availability of A_1_R was indicated by the binding potential BP_ND_ of [^18^F]-CPFPX acquired with the Logan’s reference tissue model (t* = 30 min^37^).

We reported the details of procedure and parameters with regard to PET data acquisition, preprocessing, and kinetic modeling in the method section of Ref.^27^.

#### Predicting cerebral responses by cortical and subcortical A_1_R BP_ND_

In a last step, we used A_1_R BP_ND_ to predict the GM responses identified. We first calculated the percent changes in CSR relative to BL using (meanCSR – meanBL)/meanBL × 100%. We calculated the A_1_R BP_ND_ data by cortical and subcortical regions. For cortical A_1_R BP_ND_, we averaged the values acquired in frontal, occipital, parietal, temporal, and cingulate cortices, as well as amygdala, hippocampus, and insula. For subcortical A_1_R BP_ND_, we averaged the values acquired in pallidum, olfactory tubercle, caudate nucleus, putamen, and thalamus by each hemisphere. The regions were segmented based on the automated anatomical labeling atlas (AAL^38^). We then conducted linear regression model to examine the coefficients for (1) caffeine effect, (2) interaction between caffeine effect and A_1_R BP_ND_ in predicting the percentage of the identified cerebral responses on R (R core team, Vienna, Austria). The A_1_R BP_ND_ used was taken from the hemisphere corresponding to the detected cerebral responses.

Results are presented as mean ± standard deviation.

## Main results

### Salivary concentration

On CSR Day 5, the CAFF group had a significantly higher salivary caffeine concentration than the DECAF group (CAFF: 2.24 ± 1.16 mg/L, DECAF: 0.08 ± 0.07 mg/L, t_CAFF-DECAF_ = 9.9, p < 0.001). On REC Day, the salivary caffeine concentration in the CAFF group was lower (0.19 ± 0.31 mg/L, t_CSR-REC_ = 9.7, p < 0.001) and reached a level, which did not significantly differ from the DECAF group (0.01 ± 0.02 mg/L, t_CAFF-DECAF_ = 0.8, p = 0.871).

### Multimodal analyses on grey matter

**Figure 2:**
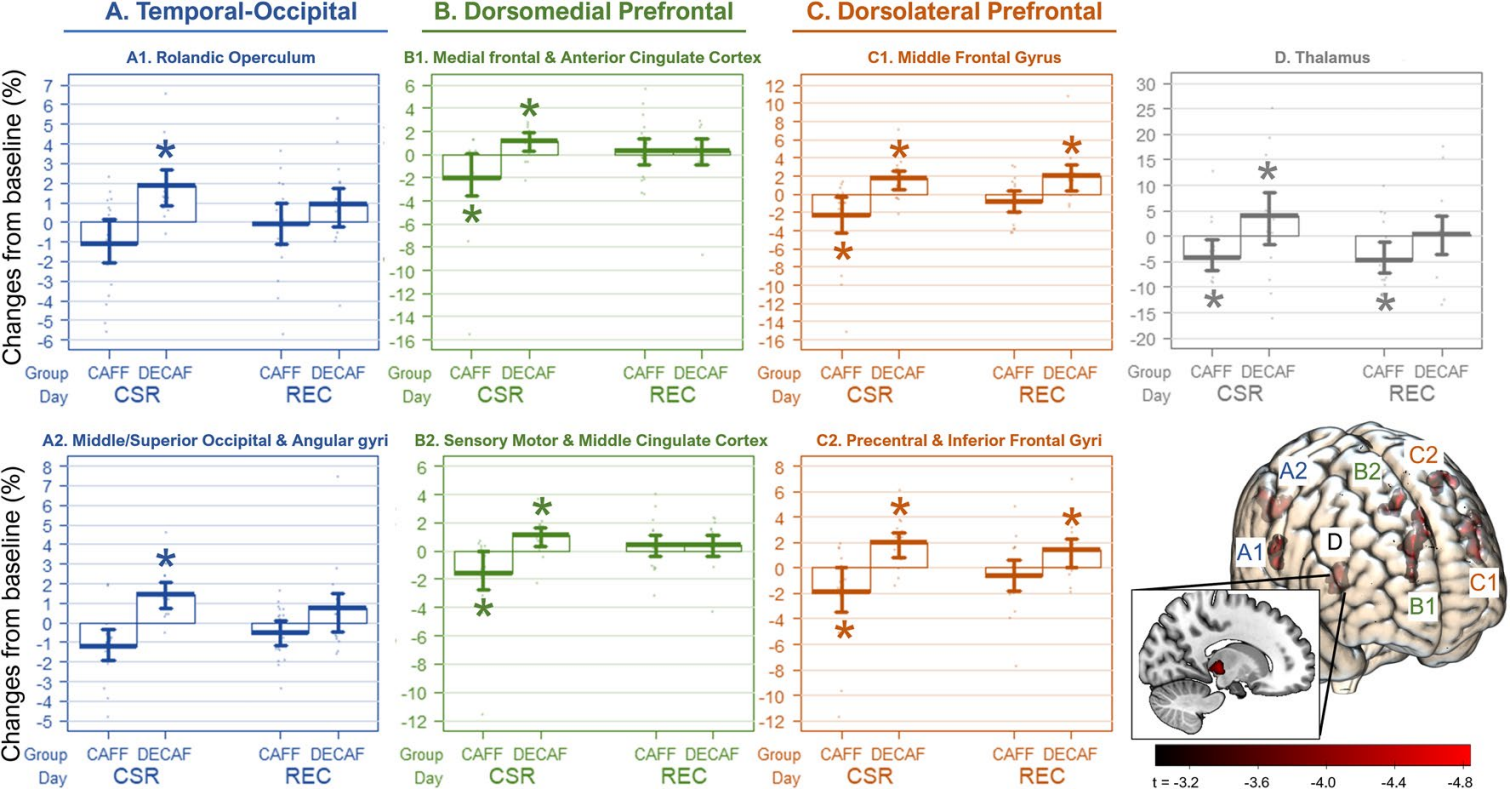
Regions showing significant differences between DECAF and CAFF groups in grey matter changes after CSR. The seven box plot panels display the clusters identified for a significant interaction effect between caffeine and CSR. The asterisks indicate a significant change compared to baseline as analyzed in post-hoc analyses. The color codes of the panels refer to a broader group of clusters based on functionality. The brain render visualizes the identified regions coded with the corresponding cluster number. The color bar indicates the t value acquired from the whole brain analysis. Finally, the render in multi-slices on the sagittal plane displays the exact location of the deeply seated Cluster D.

The results of whole-brain analyses are illustrated in Fig. 2, and the *t* values, *p* values, and Cohen’s *d* are detailed in Table 1. While no main effect of CSR on GM was found, the voxel-wise multimodal analysis controlled for the variances of CBF identified 7 large GM clusters where a significant interaction effect between caffeine and CSR was observed. The regions included temporal-occipital region: Cluster A1: *right* Rolandic operculum, Cluster A2: *right* middle occipital gyrus; dorsomedial prefrontal cortex (DmPFC): Cluster B1: *bilateral* medial superior frontal gyri and anterior cingulate cortex, Cluster B2: *left* sensory motor area and *bilateral* middle cingulate cortex; dorsolateral prefrontal cortex (DLPFC): Cluster C1: *left* middle frontal gyrus, Cluster C2: *left* precentral gyrus, inferior frontal operculum and insula; and Cluster D: *right* thalamus.

**Table 1:** Post-hoc analyses on the changes in each GM cluster after CSR and REC compared to BL by groups (CAFF N = 19; DECAF N = 17).

Cluster	Regions (N of voxels)	Group	CSR–BL			REC–BL		
			t	p_FDR_	d [95% CI]	t	p_FDR_	d [95% CI]
A. Temporal-occipital region								
A1	Rolandic Oper R (706) Postcentral R (135) SMG R (65)	DECAF	**3.9**	**0.004**	1.9 [0.7, 3.0]	1.9	0.092	0.9 [-0.1, 1.9]
		CAFF	− 2.0	0.088	− 0.9 [− 1.9, 0.0]	0.0	0.971	0.0 [− 0.9, 0.9]
A2	MOG R (827) SOG R (93) AnG R (82)	DECAF	**3.6**	**0.009**	**1.7 [0.6, 2.9]**	2.0	0.087	1.0 [− 0.0, 2.0]
		CAFF	** − 3.5**	**0.008**	** − 1.6 [− 2.6, − 0.6]**	− 0.8	0.493	− 0.4 [− 1.3, 0.5]
B. Dorsomedial prefrontal cortex								
B1	mSFG L (812) mSFG R (105) ACC L (191) ACC R (584)	DECAF	**3.1**	**0.015**	**1.5 [0.4, 2.6]**	1.1	0.329	0.5 [− 0.4, 1.5]
		CAFF	** − 2.7**	**0.018**	** − 1.2 [− 2.2, − 0.3]**	0.8	0.488	0.4 [− 0.5, 1.3]
B2	SMA L (375) MCC L (336) MCC R (186)	DECAF	**3.9**	**0.005**	**1.9 [0.7, 3.0]**	2.0	0.085	1.0 [− 0.0, 2.0]
		CAFF	** − 3.1**	**0.013**	** − 1.4 [− 2.4, − 0.4]**	1.2	0.298	0.6 [− 0.4, 1.5]
C. Dorsolateral prefrontal cortex								
C1	MFG L (506)	DECAF	**2.8**	**0.017**	**1.4 [0.3, 2.4]**	**3.2**	**0.013**	**1.6 [0.5, 2.6]**
		CAFF	** − 2.9**	**0.013**	** − 1.3 [− 2.3, − 0.3]**	− 0.8	0.513	− 0.4 [− 1.3, 0.5]
C2	Precentral L (784) IFG Oper. L (1433) IFG Tri. L (352) Rolandic Oper. L (256) Postcentral L (123)	DECAF	**4.0**	**0.009**	**1.9 [0.8, 3.1]**	**3.0**	**0.014**	**1.5 [0.4, 2.5]**
		CAFF	** − 2.5**	**0.032**	** − 1.1 [− 2.1, − 0.2]**	− 0.8	0.487	− 0.4 [− 1.3, 0.5]
D. Thalamic region								
D	Thalamus R (681) Hippocampus R (34) Lingual R (34) STG L (11)	DECAF	1.9	0.092	0.9 [− 0.1, 1.9]	0.1	0.919	0.0 [− 0.9, 1.0]
		CAFF	** − 3.0**	**0.014**	** − 1.4 [− 2.4, − 0.4]**	** − 3.0**	**0.014**	** − 1.4 [− 2.4, − 0.4]**

The d values refer to the effect size indicated by Cohen’d. The bold font indicates a statistical significance delineated by a p value < 0.05. The statistical parameters were controlled for age, sex, and total intracranial volumes in the linear mixed models. The definitions of regions are based on AAL2; voxel sizes in each region were estimated by MRIcron. All p-values provided were controlled for the false discovery rate (FDR). For additional comparison between REC and CSR, please find Supplement Table S2.

*R* right hemisphere, *L* left hemisphere, *ACC* anterior cingulate cortex, *AnG* angular gyrus, *IFG* inferior frontal gyrus, *MCC* middle cingulate cortex, MFG middle frontal gyrus, *MOG* middle occipital gyrus, *mSFG* medial superior frontal gyrus, *Oper* Operculum, *SMA* supplementary motor area, *SMG* supramarginal gyrus, *SOG* superior occipital gyrus, *STG* superior temporal gyrus, *Tri* Triangulum.

Interestingly, the post-hoc analysis revealed similarities among the different clusters in their response to CSR and recovery sleep. On CSR Day 5 compared to BL, the DECAF group exhibited a GM increase in *all* clusters. The CAFF group, however, showed a GM reduction in all clusters except *right* temporal-occipital cortex (Cluster A). On REC Day, the higher GM in the DECAF group had been remitted to the level of BL in *all* clusters except for *left* dorsolateral prefrontal cortex (Cluster C), which in general crosses the bilateral anterior and middle cingulate cortices. Furthermore, the GM reduction in the CAFF group remitted in all clusters except for thalamus (Cluster D). The response of thalamic GM inspired a supplementary correlation analysis between all the responsive regions (Supplements Fig S2), on which the following A_1_R availability analysis (see paragraph A_1_R *BP*_*ND*_* and GM plasticity*) was based.

### A_1_R BP_ND_ and grey matter plasticity

**Figure 3:**
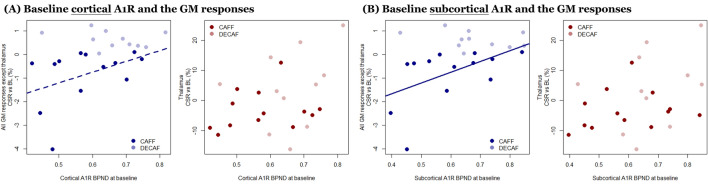
Associations between GM changes (% to baseline) and cortical/subcortical A_1_R BP_ND_. (**A**) and (**B**) Display the associations of cortical and subcortical A_1_R BP_ND_ with GM responses, respectively. The statistical parameters are derived from analyses with linear mixed models. GM changes in all regions except thalamus are in blue while changes in thalamus are in red. Dark shades represent CAFF group while lighter shades represent DECAF group. The solid line indicates a significant association between baseline A_1_R availability and the GM change, while a dash line indicates an association at trend. Find the detailed statistics from the corresponding regression analyses in Table 2.

In order to reduce the bias from multiple comparisons, we synthesized GM responses into two groups – Cluster D (thalamus) and all others – based on the intercorrelation test between the Clusters responses (Supplement Fig S2). Overall, both cortical and subcortical A_1_R BP_ND_ could partly explain the group effect on the caffeine-associated GM reduction after CSR. However, subcortical A_1_R BP_ND_ showed a stronger and significant association with the caffeine-associated GM reduction after CSR, compared to cortical A_1_R BP_ND_. Furthermore, neither cortical nor subcortical A_1_R BP_ND_ showed significant association with the GM changes in Cluster D (thalamus). The associations of cerebral responses and A_1_R BP_ND_ are presented in Fig. 3 with the corresponding stats in Table 2.

**Table 2:** Associations between GM changes (% to baseline) and cortical/subcortical A_1_R BP_ND_.

	GM in all region w/o thalamus				GM in thalamus			
(A) Baseline cortical A_1_R and the GM responses								
Model factors	β		t	p	β	t	p	
Group *CAFF—DECAF*	− 4.77		− 2.1	0.054^₸^	11.50	0.4	0.667	
Cortical BP_ND_ *in CAFF*	4.55		2.0	0.065^₸^	12.84	0.5	0.631	
Cortical BP_ND_ *in DECAF*	− 0.10		− 0.4	0.728	39.72	1.2	0.227	
(B) Baseline subcortical A_1_R and the GM responses								
Model factors		β	t	p	β	t		p
Group *CAFF—DECAF*		− 4.96	− 2.8	0.012	9.16	0.4		0.677
Subcortical BP_ND_ *in CAFF*		4.71	2.7	0.015	10.09	0.5		0.640
Subcortical BP_ND_ *in DECAF*		− 0.99	− 0.5	0.641	33.14	1.3		0.204

Table (A) and (B) present the statistics of the linear model examining the explanatory power (β) of the main effect of group (CAFF vs DECAF; row 1) and the interaction effect between group and BP_ND_ (row 2 & 3) on the variance of regional GM. Besides a significant group effect on all GM regions (row 1), we further observed an interaction effect between Group and A_1_R BP_ND_, namely that an association between the cortical (at trend)/subcortical BP_ND_ (significant) with all GM regions except for thalamus specifically in the CAFF group (row 2). The associations between regional GM and cortical/subcortical BP_ND_ are visualized as Fig. 3 (A) and (B) respectively. Underscored p values were emphasized for the statistical significance, while the ^₸^Mark refers to the effects that showed changes at trend.

### Exploratory analysis on cerebral blood flow

The whole-brain analysis on CBF did not indicate a significant difference between CSR Day 5 and BL Day or an interaction of caffeine with condition (i.e. CSR vs. BL)-BL. However, based on the solid evidence on the effects of caffeine and caffeine cessation on reducing and elevating CBF, respectively^9,39–46^, we examined the GM changes from CSR to REC, as well as a group difference in such changes. Interestingly, we found a significantly lower CBF on CSR compared to REC Day in the cerebellum, pons, brain stem, thalamus, hypothalamus, inferior temporal gyrus, and prefrontal cortex (all regions: p_FDR-corrected_ < 0.05; Supplements Fig S3); however, we did not find a significant group difference in the divergent responses between CSR and REC. To provide further information, we conducted a series of supplementary analyses on the effect of CSR or REC compared to BL Day with a lower statistical threshold specifically in the regions showing lower CBF on CSR Day than REC Day (i.e. the medial frontal cortex, subcortical regions, occipital cortex, cerebellum, and midbrain). We found a reduced CBF on the CSR Day and an elevated CBF on REC Day compared to the BL Day in the CAFF group. The statistics and figures are presented in the Supplements Fig S3.

## Discussion

The current study examined the cortical plasticity in response to CSR with or without the concomitant interference of caffeine. Independent of the caffeine-induced variances in CBF, we found opposite GM responses between the caffeine and decaffeinated groups after CSR. Specifically, GM in the left DLPFC, as well as right DmPFC, temporal-occipital, and thalamic cortices were increased after 5-day CSR without caffeine; in contrast, concomitant caffeine intakes during CSR led to a decrease in prefrontal and thalamic cortices. All the cortical changes, except for the DLPFC increase in the DECAF group and the thalamic decrease in the CAFF group, recovered to the baseline level after an 8-h sleep opportunity (which included an approximately 35 h withdrawal from caffeine for the CAFF group). Most importantly, individuals with a lower availability of subcortical A_1_R at baseline showed a larger caffeine-associated GM decrease after CSR in all responsive regions except for thalamus. Taken together, our data reveal region-dependent GM plasticity after CSR, which diverges between the presence and absence of caffeine. Furthermore, individual trait in A_1_R availability may play a resilient role against the effects of caffeine on GM changes after CSR.

### Implications on use-dependent cortical and synaptic plasticity associated with adenosine systems

#### GM plasticity and synaptic strengths

Brain structural alterations induced by sleep loss have been frequently reported. Extensive sleep loss such as 36-h to 72-h sleep deprivation^7,8^ as well as one month of CSR^15^ was found to lead to reduced GM or structural network. Moreover, 21-h extended wakefulness followed by 3-h time in bed resulted in both reduced GM in thalamus, precuneus, and precentral gyrus as well as increased GM in insula^6^. Our data showing increased GM in DLPFC, DmPFC, thalamus, and temporal-occipital regions after 5 days of CSR with 5-h time in bed daily seemingly conflicts to the GM reduction commonly observed. However, the inconsistency may lie in the subtle differences in the sleep protocols – specifically, the total days of sleep restriction and the different amounts of sleep as daily recovery process. The synaptic homeostasis hypothesis (SHY)^47^ elaborated how synaptic homeostasis is maintained in sleep–wake cycles. It is suggested that the synaptic strengths are increased and saturated throughout wakefulness and downregulated as a restorative process during sleep^47^. Extensive sleep loss, therefore, may instead lead to neuronal exhaustion and hence the commonly observed GM reduction. However, partial sleep (i.e. 5-h time in bed) may allow partial brain restoration, which enables the synaptic strengths to maintain its upregulated state without an exhaustion within limited days of sleep restriction, therefore an increased GM in the current study. An earlier study examining the trajectory of GM changes throughout a 36-h sleep deprivation found a non-linear course of responses^7^. They observed increased GM in striatum and cingulate cortices throughout 36-h sleep deprivation, while a regional GM reduction only started to show at 32 h onward in the thalamus, insula, and somatosensory association cortex^7^. Their findings support the adaptability of brain structure to a certain extent of sleep deprivation until exhaustion might occur. On the contrary, caffeine has been shown to reduce the synaptic long-term potentiation (LTP) in rodents^48^ and LTP-like cortical activity in humans^49^, which may counteract the sleep loss-induced brain plasticity. In other words, the saturation and suppression of synaptic strengths might underlie the CSR-associated GM increase and caffeine-associated GM decrease, respectively.

#### Adenosine system in energy use-dependent synaptic and cortical plasticity

GM plasticity may occur in response to an increased or decreased need for energy resources, for which adenosine is a critical index as a byproduct of energy usage^20,21^. A study found a higher GM volume in the morning compared to the evening^50^ and suggested that the time of day-dependent plasticity of GM may be an evolutionary adaption to the energy consumption of diurnality^50^. In sleep homeostasis process, the elevated and reduced adenosinergic activity throughout wakefulness and sleep, respectively, is believed to be a biological index of the accumulation and dissipation of homeostatic sleep pressure^21^. By blocking adenosine receptors, caffeine counteracts the increased sleepiness as one of the classic effects and switches on the “energy saving mode” by reducing cortical activity^51^, cerebral metabolic rate of glucose^52^, and cerebral metabolic rate of oxygen (CMRO2)^53,54^. Our data show that caffeine does not only counteract the CSR-associated GM increase but further downregulate cortical intensity potentially through hindering the adenosine-mediated energy consumption, which is in line with earlier evidence of a reduced or lower GM volume after 10-day 450 mg caffeine intake^9^ or in habitual high-dose caffeine consumers^10^ in various brain regions.

The association between striatal A_1_R availability and cortical reduction after CSR with caffeine implicates a role of the adenosine system in the CSR- and caffeine-associated GM plasticity. Specifically, a higher A_1_R availability seems to serve a function of resilience against caffeine-associated GM reduction. Such an A_1_R-dependent resilience has also been found to be against cognitive impairments induced by sleep deprivation^55^. Interestingly, despite the cortical and subcortical A_1_R availability being highly correlated, we observe a much stronger association of caffeine-related GM reduction with subcortical than cortical A_1_R availability. In striatum, A_1_Rs antagonistically interact with A_2A_R through a formation of G-protein coupled-receptor heterodimer as a “*concentration-dependent switch*”^56^ that modulates presynaptic glutamate release based on the extracellular adenosine levels. In a regular extent of wakefulness, the tonic neuro-inhibition maintained by A_1_R is strengthened as the waking state lasts longer, leading to a reduced striatal presynaptic glutamate release and neural firing^57,58^. This effect is likely to underlie the lower GM in the evening compared to mornings^50^. In relatively shorter (e.g. 12 h or 24 h) sleep deprivation^7^ and sleep restriction, adenosine may be rapidly elevated^22^, triggering the phasic excitatory adenosine A_2A_ receptors, which in turns inhibits A_1_R as well as enhances glutamate release^56,59,60^ and neural plasticity^24,61,62^, there by potentially increasing GM as observed in the DECAF group. However, a prolonged neuroexcitation by an extensive sleep loss, e.g. a 36-h or 72-h deprivation or one-month sleep restriction, might turn into a detrimental hyperexcitability^63^ and lead to a decreased brain structure^7,8,15^. Caffeine, on the other hand, acutely enhances striatal glutamate release by its predominant antagonism at A_1_R^64^. However, daily or chronic caffeine administration diminishes the effect of A_1_R antagonism on increasing glutamate level^65^, which can lead to suppressing LTP and neuroplasticity^48,49^, thereby potentially a reduced GM as observed in the CAFF group^9,10^. Furthermore, through a gradually reduced affinity of A_2A_R to caffeine over daily or chronic intake^56,60^, a concomitant exposure to high extracellular adenosine level such during CSR allows the A_2A_R rebinding to the endogenous adenosine even with the presence of caffeine, thereby regaining the inhibition to A_1_R through the A_1_R-A_2A_R heterodimers in striatum^56,60^. The association between striatal A_1_R availability and caffeine-associated GM reduction after CSR further implicate a role of A_1_R in synaptic homeostasis and GM plasticity during caffeine intake and sleep loss.

### Beyond the use-dependent perspective: unique GM response in thalamus

Interestingly, although thalamic GM shows a consistent CSR-associated GM increase and caffeine-associated GM decrease, its recovery seems to be much slower than other responsive regions. An earlier study following up the trajectory of medial temporal GM recovery after the cessation of 10-day 450 mg caffeine intake suggested a recovery time at minimum 36 h^44^. The current study using a lower caffeine dose with a shorter intake period found a shorter time needed for a full recovery for most of the responsive regions, except for thalamus. Furthermore, the GM reduction in thalamus is not directly associated with the individual A_1_R availability. An earlier study found regional differences in the change in adenosine signaling after sleep deprivation^66^. While adenosine was rapidly accumulated and dissipated in basal forebrain and cortices after sleep deprivation and during recovery sleep, in thalamus, together with midbrain regions, showed a continuous decrease in adenosine concentration after sleep deprivation and even a few hours into recovery sleep^66^. Such differences in adenosinergic activity between thalamus and other brain regions might underlie the unique responses of thalamus in our data. Since thalamus is one of the most critical regions in sleep–wake regulations^67,68^, and the structural or functional alterations of thalamus are often associated with insomnia^69–74^ or hypersomnia^75^, it may be impacted by the concomitant CSR and caffeine intake in a more critical manner compared to other cortical regions.

### Limitations and summary

A few limitations in this study require careful interpretation and further investigation in the future. First, although we preselected participants based on the *ADORA2A* polymorphism, the lack of A_2A_R availability data as derived from PET precludes a broader understanding of the receptor’s role in sleep loss induced GM changes. This limitation warrants more adenosine and A_2A_R-focusing studies on the effects of CSR and daily caffeine intake to be conducted. Second, it might be argued that the estimation of GM by MRI T1-weighted image can be potentially impacted by caffeine-induced changes in perfusion^30^. However, our study included both structural imaging and CBF measurement, and we did not observe associations between the two, including the pattern of changes across regions. An earlier study further suggests that the covariance between T1-weighted measurement and CBF rather occurs on a global instead of regional level^9^. Lastly, the caffeine and decaffeinated treatments in the current study were delivered as identically brewed coffee only different in the caffeine content, which indeed guaranteed the precision of caffeine administration. However, with other ingredients contained in brewed coffee, the observed outcomes could not be fully attributed to the effects of caffeine alone, but it could also be an effect from the interaction between caffeine and other biochemicals. Nevertheless, using brewed coffee is preferred given that it provides a better generalizability to a real-life coffee intake.

In summary, this study revealed reversible cortical plasticity in frontal, temporal-occipital, and thalamic GM in response to CSR. This plastic response, however, can be suppressed or reversed by concomitant caffeine intake. The individual baseline subcortical A_1_R availability may play a role in the caffeine-associated GM response. More studies are warranted to further investigate the role of A_2A_R in these GM changes.

### Supplementary Information

Supplementary Information.

## Data Availability

All the data reported in this manuscript is available for research purposes upon request. Please contact d.elmenhorst@fz-juelich.de for the request.
