# Supplementary material for: Repeated caffeine intake suppresses cerebral grey matter responses to chronic sleep restriction in an A1 adenosine receptor-dependent manner: a double-blind randomized controlled study with PET-MRI
Source: Sci Rep. 2024 Jun 3;14:12724. doi: 10.1038/s41598-024-61421-8 (PMC11148136; doi:10.1038/s41598-024-61421-8)
Supplement: Supplementary file 1 — Supplementary Information. [file 41598_2024_61421_MOESM1_ESM.pdf]

Supplement

I. Supplementary methods

1. Inclusion/Exclusion criteria

Inclusion criteria

- Females or males
- 20 to 40 years old
- ADORA2A c.1976 C / C , C / T, and T / T allele carrier in decreasing order of priority
- no sleep disturbances
- normal chronotype
- non-smoker
- average caffeine consumption
- 2-week abstinence before start of the study
- presence of a written informed consent
- women in a reproductive age a negative pregnancy test and an effective contraception for at least 3 months

Exclusion criteria

- BMI > 30
- missing of a legally effective informed consent
- not able to speak German
- any medication which influences research Parameters
- blood-thinning medication or known coagulation disorder
- chronic diseases: e.g. neurological, cardiopulmonary, endocrinological, renal, hepatic, psychiatric, autoimmune diseases; sleep disturbances
- shift or night work
- cumulative or professional radiation exposure
- drug dependence, smokers; electric implants for stimulation or therapy
- metallic objects in or on the body (e.g. surgical screws) without MRI-compatibility
- fixed braces or retainer
- tattoos in the region under Investigation
- intense grinding operations on metallic objects in the month ago
- Medical complaints that prevent subjects from lying still, e.g. back pain
- pregnancy, breast feeding
- people not qualified for legal acts or in custody
- other condition which, according to the medical doctors involved, makes someone unsuited for participating in the study (e.g. claustrophobic)
- copper spiral
- contraceptive coil interacting with MRI
- Epworth Sleepiness Scale (ESS) > 10, Apnea Hypopnea Index (AHI)> 10, Periodic Leg movements (PLMS)> 15

2. Validation of Voxelstats toolbox using FSL as reference method

Fig S1. Validation of the novel toolbox and the voxel-to-voxel approach in tackling the bias of perfusion on morphometry. We used the "*randomise*" function in FMRIB Software Library (FSL 5.0; Oxford Center for Functional MRI of the Brain, United Kingdom) as a reference and examined the interaction effect between caffeine and CSR on GM controlling for *global* CBF. For both analyses, we used age, sex, and total intracranial volume as regressors as covariates, and the number of permutations in both analyses was set at 5000. The statistic maps derived from FSL using global as covariate (A) and from VoxelStats using voxel-to-voxel approach (B) are rather consistent.

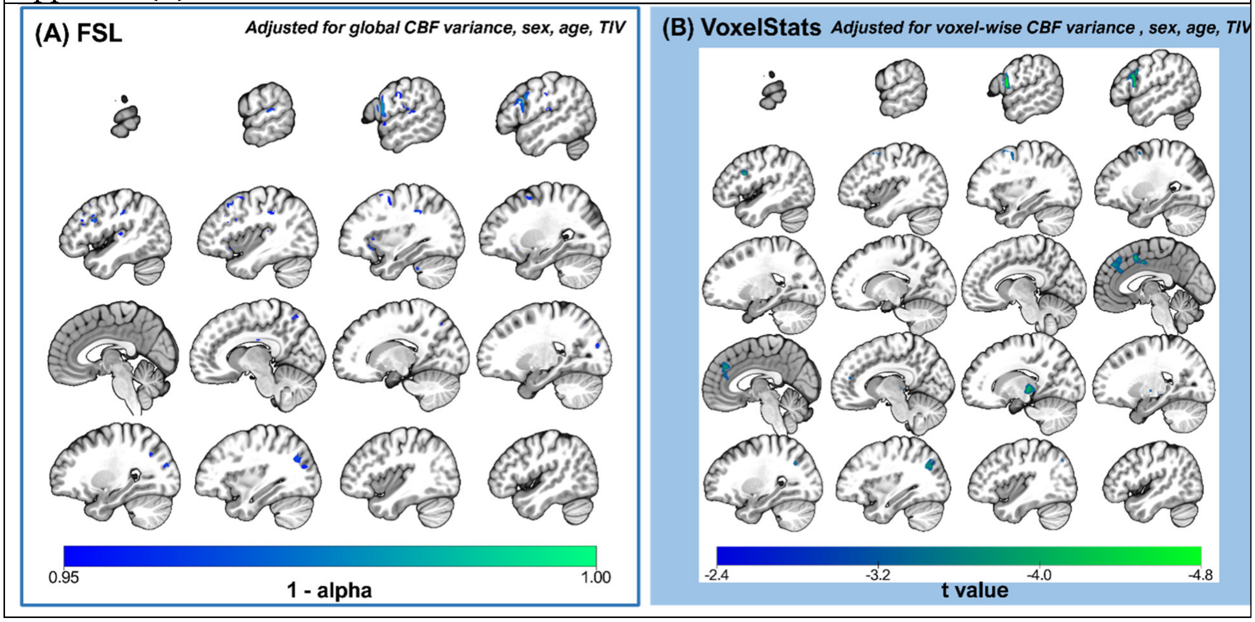

II. Supplementary data

Table S1. Demographic descriptions by groups

	CAFF group	DECAFF group	Statistics	p-value
**N**	**19**	**17**	**χ^2 = 0.1**	**0.739**
Age	29.9 ± 5.0	27.8 ± 5.3	**t = 1.2**	**0.230**
Sex (F:M)	8:11	7:10	**χ^2 = 1.0**	**0.800**
Genotype (C.C./C.T/T.T)	14/4/1	15/2/0	**χ^2 =3.8**	**0.574**
BMI	23.1 ± 2.8	23.4 ± 2.3	**t = 0.3**	**0.735**
Habitual caffeine intake **(mg)**	150.3 ±128.1	109.1 ± 92.1	**t = 1.1**	**0.281**

III. Supplementary results

Figure S2. Inter-correlation between Clusters. The cross-correlation was analyzed and plotted using R package *corrplot (https://github.com/taiyun/corrplot)*. Panel (a) and (b) indicate the correlation between GM responses (i.e. changes from baseline) in CAFF and DECAF groups on CSR Day, respectively. The color bars below indicate the Pearson's correlation coefficient. The results of the cross-correlation suggest a uniquely weaker relationship between Cluster D (thalamus) and other clusters, and the discrepancy was particularly large in CAFF compared to DECAF group. This supplementary analysis was used to identify the similarities among GM responses and thereby group the regional data for further analysis.

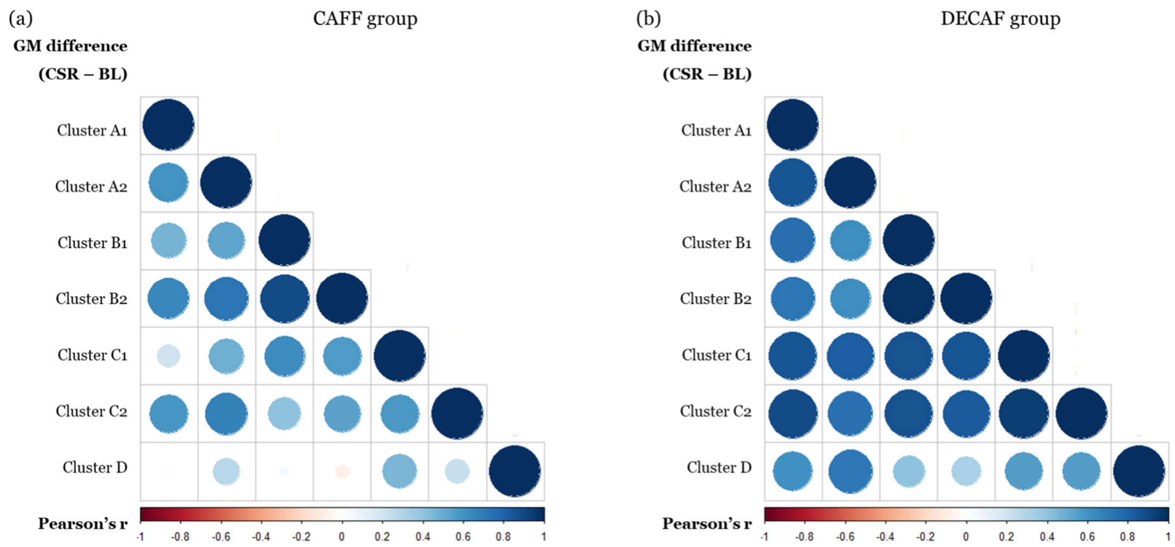

Figure S3. Supplementary analysis on the interaction effect of Caffeination x Day on CBF. Panel (a): Regions that showed significant difference in CBF between CSR vs REC. The color bar refers to (1 – p value), i.e., the brighter the color is, the stronger the statistical significance is stated. Panel (b): the mean CBF response across the responsive region (CSR vs REC) on each day in each group. Supplementary analysis: In light of the contrast between CSR vs REC, we assumed an underpower issue and therefore attempted to provide more information on the trend of the CBF changes by lowering the statistical threshold ($P_{FDR\text{-}corrected} < 0.20$ but > 0.05). We expected to find a reduced CBF on the CSR Day and an increased CBF on the REC Day compared to BL. The voxel-wise whole-brain analysis with the reduced statistical threshold indicated an interaction effect between Caffeination x CSR in the regions corresponding to the contrast of CSR vs REC, i.e. the medial frontal cortex, subcortical regions, occipital cortex, cerebellum, and midbrain. The linear mixed model on the extracted CBF response indicated a significantly stronger reduction of CBF on the CSR ($t_{interaction} = -3.2$, $p_{interaction} = 0.002$) and elevation on REC Day ($t_{interaction} = 3.4$, $p_{interaction} = 0.001$) in the CAFF group compared to the DECAF group.

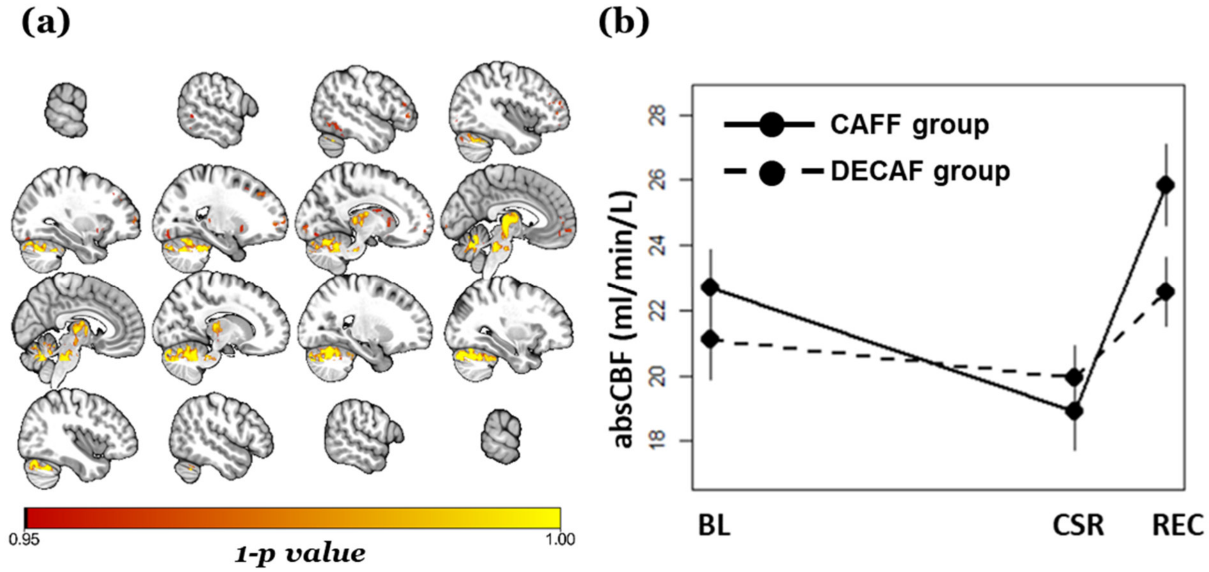

Table S2. Supplementary analysis on the GM differences between REC and CSR by groups (CAFF N=19; DECAF N=17). The d values refer to the effect size indicated by Cohen'd. The bold font indicates a statistical significance delineated by a p value < .05. The statistical parameters were controlled for age, sex, and total intracranial volumes in the linear mixed models. The definitions of regions are based on AAL2; voxel sizes in each region were estimated by MRIcron. The details of statistical methods and abbreviations, please refer to the ***Method*** and ***Table 1*** in the main manuscript.

			REC – CSR		
Cluster	Regions (N of voxels)	**Group**	**t**	**p**	**d [95% CI]**
**A. Temporal-Occipital region**					
A1	Rolandic Oper R (706)<br>Postcentral R (135)<br>SMG R (65)	**DECAF**	-2.0	0.056	-1.0 [-2.0 – 0.0]
		**CAFF**	**2.0**	**0.050**	**1.0 [0.0 – 1.9]**
A2	MOG R (827)<br>SOG R (93)<br>AnG R (82)	**DECAF**	-1.6	0.123	-0.8 [-1.8 – 0.2]
		**CAFF**	**2.7**	**0.010**	**0.7 [-0.2 – 1.7]**
**B. Dorsomedial prefrontal cortex**					
B1	mSFG L (812)<br>mSFG R (105)<br>ACC L (191)<br>ACC R (584)	**DECAF**	-1.9	0.064	-1.0 [-2.0 – 0.0]
		**CAFF**	**3.6**	**0.001**	**1.8 [0.7 – 2.8]**
B2	SMA L (375)	**DECAF**	-1.9	0.063	-1.0 [-2.0 – 0.0]

	MCC L (336)<br>MCC R (186)	**CAFF**	**4.5**	**<0.001**	**1.8 [0.7 – 2.8]**
**C. Dorsolateral prefrontal cortex**					
C1	MFG L (506)	**DECAF**	0.4	0.690	0.2 [-0.8 – 1.1]
		**CAFF**	**2.2**	**0.036**	**1.0 [0.1 – 2.0]**
C2	Precentral L (784)<br>IFG Oper. L (1433)<br>IFG Tri. L (352)<br>Rolandic Oper. L (256)<br>Postcentral L (123)	**DECAF**	-1.0	0.332	-0.5 [-1.4 – 0.5]
		**CAFF**	1.7	0.090	0.8 [-0.1 – 1.8]
**D. Thalmaic region**					
D	Thalamus R (681)<br>Hippocampus R (34)<br>Lingual R (34)<br>STG L (11)	**DECAF**	-1.8	0.083	-0.9 [-1.9 – 0.1]
		**CAFF**	-0.1	0.956	0.0 [-0.9 – 0.9]
